# Vitamin D, Vitamin D-Binding Proteins, and VDR Polymorphisms in Individuals with Hyperglycaemia

**DOI:** 10.3390/nu14153147

**Published:** 2022-07-30

**Authors:** Rajiv Erasmus, Setjie Maepa, Ian Machingura, Saarah Davids, Shanel Raghubeer, Tandi Matsha

**Affiliations:** 1 Department of Pathology, Chemical Pathology Division, National Health Laboratory Service (NHLS) and Stellenbosch University, Tygerberg Hospital, Cape Town 7505, South Africa; rte@sun.ac.za (R.E.); setjiew@gmail.com (S.M.); 2 SAMRC/CPUT/Cardiometabolic Health Research Unit, Department of Biomedical Science, Faculty of Health & Wellness Sciences, Cape Peninsula University of Technology, Cape Town 7530, South Africa; ianmachingura@gmail.com (I.M.); davidss@cput.ac.za (S.D.); matshat@cput.ac.za (T.M.)

**Keywords:** hyperglycaemia, South Africa, vitamin D, vitamin D receptor, vitamin D-binding protein

## Abstract

Vitamin D reportedly plays an important role in the pathogenesis of diabetes mellitus; however, this role is unclear and debated. This study investigated the association between 25(OH) vitamin D, vitamin D-binding proteins, and vitamin D receptor (VDR) polymorphisms in healthy individuals and those with prediabetes and type 2 diabetes mellitus (T2D) from South Africa. A cross-sectional study was conducted involving subjects of mixed ancestry aged ≥20 years. Males presented with higher mean 25(OH) vitamin D levels than females, while females exhibited significantly higher serum vitamin D-binding protein levels. Significant differences in mean 25(OH) vitamin D levels were observed in normo-glycaemic, prediabetes, screen-detected DM, and known DM individuals. Vitamin D receptor SNPs *Fok1* and *Taq1* were not associated with glycaemic status. *Fok1* was not associated with 25(OH) vitamin D deficiency, while *Taq1* was associated with vitamin D insufficiency. This study showed a high prevalence of vitamin D deficiency/insufficiency in this South African population, with decreased vitamin D levels observed in hyperglycaemic individuals, which was not linked to either vitamin D-binding protein or polymorphisms in *Fok1* of the *VDR* gene. These results may be used as a platform for further research into diagnosis and treatment of hyperglycaemia.

## 1. Introduction

The role of vitamin D in non-communicable chronic diseases and the potential impact of vitamin D supplementation as a preventive and therapeutic measure is controversial and debated [1,2,3,4]. In cross-sectional epidemiological studies, insufficient vitamin D status is associated with the development of obesity, metabolic syndrome, and type 2 diabetes (T2D) in several but not all reports [5,6,7,8]. A recent systematic review reported that vitamin D supplementation at a minimum dose of 100 µg/d (4000 IU/d) may significantly reduce serum fasting plasma glucose (FPG), glycosylated haemoglobin (HbA1c), and homeostatic model assessment of insulin resistance (HOMA-IR) index in type 2 diabetic patients [9]. However, in a randomized trial, Pittas et al. concluded that vitamin D supplementation does not lower the risk of T2D [8]. Recently, Pilz et al. critically appraised several vitamin D randomized controlled trials and suggested that many researchers should carefully investigate the cohorts included in these studies, as cohort choice may bias the results obtained during these studies [10]. Furthermore, Xu et al. reported on a greatly improved sample size and concluded that genetically increased vitamin D concentration decreased T2D risk, suggesting that vitamin D supplementation deserves further investigation in interventional studies [11].

Vitamin D-binding protein is a serum glycoprotein, which is the major carrier protein of vitamin D sterols, and is essential for the intracellular metabolism of vitamin D. Variations in vitamin D-binding proteins are postulated to influence the amount and activity of vitamin D, which in turn affect insulin secretion, β-cell dysfunction, and glucose metabolism [12]. Vitamin D exerts its effects on target tissues by binding to the cytosolic/nuclear vitamin D receptor (VDR), a member of the steroid/thyroid hormone receptor family. The VDR is expressed in the pancreas, and four polymorphisms of the vitamin D receptor, namely *FokI*, *BsmI*, *ApaI*, and *TaqI*, have been identified to be associated with insulin secretion and sensitivity. However, some studies have found no associations with these polymorphisms [13].

The mixed-ancestry population of Bellville South, Cape Town, exhibits a high prevalence of T2D [14], and vitamin D deficiency may contribute to the high prevalence of diabetes observed in this population group. In this study, we investigated the association between 25(OH) vitamin D levels, vitamin D-binding protein, and VDR polymorphisms in subjects with prediabetes and T2D in a mixed-ancestry South African population, as research is lacking in this population group.

## 2. Materials and Methods

### 2.1. Ethical Approval

The study forms part of the ongoing Vascular and Metabolic Health study (VMH), which received ethical approval from the research ethics committees of the Cape Peninsula University of Technology (CPUT) and Stellenbosch University (NHREC: REC-230 408–014 and N14/01/003, respectively). The current study also received ethical approval from the Stellenbosch University Health Research Ethics committee (0719) and Cape Peninsula University of Technology, Faculty of Health and Wellness Sciences Research Ethics committee (CPUT/HW REC2015/H01). Written informed consent was sought from all study participants following explanation of study procedures in their language of choice. All methods were performed in accordance with the Declaration of Helsinki and all relevant regulations. 

### 2.2. Study Population and Design 

This cross-sectional study comprised 1989 participants of mixed ancestry aged ≥ 20 years residing in Bellville South, Cape Town, South Africa. For the final analysis, individuals with incomplete data, acutely ill individuals, and pregnant females were excluded from the study. Therefore, the final sample size for this study was 968, from a total of 1989 participants. A detailed description of the survey and procedures conducted in this study have been published [14].

### 2.3. Clinical Data 

The demographic and clinical data have been previously described, and were collected using a questionnaire [15]. The weight was measured to the nearest 0.1 kg using the Omron body fat meter HBF511 digital bathroom scale with participants wearing light clothing without shoes. The stadiometer was used to measure body height to the nearest centimetre with the study participants standing on a flat surface. The body mass index was calculated as weight per square meter (kg/m^2^). The waist circumference was measured using a non-elastic tape at the level of the narrowest part of the torso, as seen from the anterior view, whilst in obese participants the narrowest circumference between the ribs and the iliac crest were measured. Hip circumference was measured at the maximal circumference over the buttock using a non-elastic tape. Body mass index was used to classify participants as underweight (˂18.5 kg/m^2^), normal (18.5–24.99 kg/m^2^), overweight (≥25 kg/m^2^), or obese (≥30 kg/m^2^) according to the World Health Organisation (WHO) criteria [16]. Participants who did not have a medical history of diagnosis with diabetes mellitus underwent a 2 h oral glucose tolerance test (OGTT), as recommended by the WHO. The OGTT was used to group the study participants as normoglycaemic or prediabetic (including impaired fasting glycaemia, impaired glucose tolerance, or a combination of both) using the WHO criteria [17]. The Joint Interim Statement of the International Diabetes Federation Task Force on Epidemiology and Prevention, National Heart, Lung and Blood Institute, American Heart Association, World Heart Federation, International Atherosclerosis Society, and International Association for the Study of Obesity (JIS) was used to classify metabolic syndrome (MetS) [18].

### 2.4. Biochemical Analysis

Blood samples were collected from all participants after fasting overnight. Plasma glucose was measured using the hexokinase method (Cobas 6000, Roche Diagnostics; Mannheim, Germany), HbA1c using high-performance liquid chromatography (HPLC; Bio-Rad Variant Turbo, Bio-Rad, South Africa), which was National Glycohaemoglobin Standardization Program (NGSP) certified, insulin using the paramagnetic particle chemiluminescence assay (Beckman DXI, Beckman Coulter, South Africa), fructosamine using a colorimetric test with nitro blue tetrazolium (Cobas c311, Roche Diagnostics), low-density lipoprotein cholesterol (LDL-C; mmol/L) using enzymatic selective protection-end point (Beckman AU, Beckman Coulter), HDL-C (mmol/L) using enzymatic immune-inhibition—end point (Beckman AU), and triglycerides (TG; mmol/L) using glycerol phosphate oxidase-peroxidase—end point (Beckman AU). The 25(OH) vitamin D levels were measured using the paramagnetic particle chemiluminescence test (Beckman DXI), and vitamin D-binding protein (VDBP) was determined using the Human Vitamin D BP Quantikine ELISA kit (DVDBP0; R&D Systems, Minneapolis, MN, USA).

### 2.5. Definition of Vitamin D Deficiency

Vitamin D deficiency was defined using either the 2011 Endocrine Clinical Society Practice Guidelines as 25 hydroxyvitamin D (25(OH) vitamin D) below 20 ng/mL (50 nmol/L) and vitamin D insufficiency as 25(OH) vitamin D between 20 and 29 ng/mL (50–75 nmol/L) [19] or the Global Consensus Recommendations on Prevention and Management of Nutritional Rickets, with vitamin D deficiency defined as 25(OH) vitamin D below 12 ng/mL (30 nmol/L) and vitamin D insufficiency as 25(OH) vitamin D between 12 and 20 ng/mL (30–50 nmol/L) [20].

### 2.6. Genetic Analysis

Genomic DNA was extracted from whole blood samples collected in EDTA tubes using the salt extraction method, then quantified using the NanoDrop ND-1000 instrument (Nanodrop Technologies, Wilmington, USA). *VDR* single-nucleotide polymorphisms, *Fok1* (*rs2228570*), *Apa1* (*rs7975232*), and *Taq1* (*rs731236*), were genotyped using high-throughput real-time polymerase chain reaction on the Bio-Rad Optica platform (Bio-Rad, Hercules, CA, USA) using TaqMan™ genotyping assays. Primers were predesigned TaqMan™ SNP genotyping assays. All primers and kits comply with the minimum information for publication of quantitative RT-PCR experiments (MIQE). All primer sequences can be accessed via the Thermo Fisher Scientific (Waltham, MA, USA) website. Thereafter, all samples were submitted to Inqaba Biotechnical Industries (Pretoria, South Africa) for further verification by an independent laboratory. The conventional polymerase chain reaction followed by direct DNA sequencing was performed for analytical validation of genotyping.

### 2.7. Data Analysis

Data were analysed using Statistica 13.3 (StatSoft, Pretoria, South Africa). Categorical variables were summarized as count and percentages, while quantitative variables were indicated as mean (standard deviation) or median (25th–75th percentiles). Variable comparisons across the glycaemic status were conducted using the chi-squared test. The Pearson chi-square test was used to determine association between single-nucleotide polymorphism genotypes and/or allele frequencies and vitamin D deficiency, obesity, and glycaemia categories. A multiple linear regression model was used to establish possible associations between vitamin D and other test results. A *p*-value < 0.05 was considered statistically significant.

## 3. Results

### 3.1. Participant Characteristics and Vitamin D Deficiency

A total of 968 study participants were recruited, 79.2% of which were female. Table 1 indicates the characteristics of study participants and vitamin D categories (deficiency, insufficiency, and optimal) using the 2011 Endocrine Clinical Society Practice Guidelines (*p* = 0.004) and the Global Consensus Recommendations on Prevention and Management of Nutritional Rickets (*p* = 0.003), varied according to sex. Male participants were older, exhibited greater waist/hip ratios and higher glucose/insulin ratios and levels of serum creatinine, urine creatinine, aspartate aminotransferase, serum albumin, urine sodium, and cotinine. Female participants exhibited greater waist and hip circumference measurements, higher levels of two-hour postprandial blood glucose, HbA1c, fasting blood insulin, two-hour postprandial blood insulin, HDL cholesterol, LDL cholesterol, cholesterol, parathormone, calcium corrected, and phosphate, and included a greater percentage of smokers. Vitamin D and vitamin D-binding protein levels varied according to sex, with males exhibiting higher mean 25(OH) vitamin D levels than females (24 ± 8 vs. 22 ± 8 ng/mL, *p* = 0.0006, respectively), whilst females displayed significantly higher serum vitamin D-binding protein levels (323 ± 81 vs. 306 ± 74 µg/mL, *p* = 0.007).

Using the 2011 Endocrine Clinical Society Practice Guidelines, the prevalence of vitamin D deficiency and insufficiency amongst all participants was 44.5% and 42.5%, respectively. As expected, when using the Global Consensus Recommendations on Prevention and Management of Nutritional Rickets, the prevalence of vitamin D deficiency and insufficiency amongst all study participants was 5.6% and 39.2%, respectively (Table 2). The Institute of Medicine (IOM) recommended a minimum vitamin D level of ≥20 ng/mL for adults in 2011; thus, the mean vitamin D level amongst all study participants fell within this recommended level [1,21].

### 3.2. Glycaemic Status and Vitamin D Deficiency

Table 3 shows the characteristics of the study participants according to their glycaemic status. Using the 2011 Endocrine Clinical Society Practice Guidelines for vitamin D deficiency, vitamin D varied according to the glycaemic status, with normoglycaemic participants displaying higher vitamin D levels than prediabetes mellitus, screen-detected diabetes mellitus, and known diabetes mellitus participants (*p* = 0.002).

**Figure 1:**
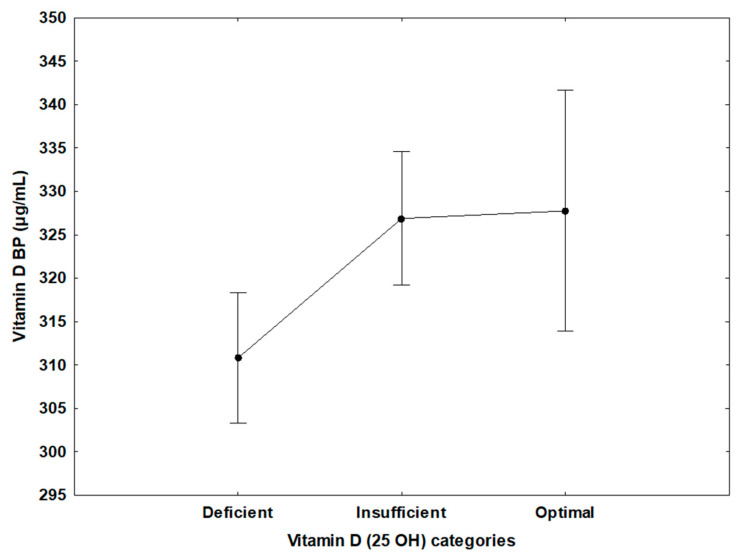
Using the Endocrine Clinical Society Practice Guidelines for vitamin D deficiency, vitamin D-binding protein (µg/mL) levels were found to be significantly decreased in the deficient vitamin D group, mean ± SD, 311 ± 75 (µg/mL) (*N* = 431), compared to the insufficient vitamin D group, 327 ± 85 (*N* = 408), and the optimal vitamin D group, 328 ± 75 (*N* = 126), (*p* = 0.007). BP, binding protein.

**Figure 2:**
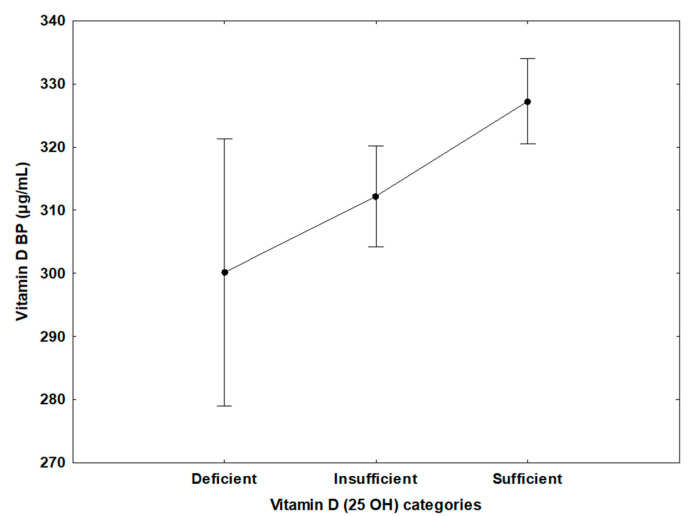
Using the Global Consensus Recommendations on Prevention and Management of Nutritional Rickets vitamin D deficiency, vitamin D-binding protein (µg/mL) levels were found to be significantly decreased in the deficient vitamin D group, mean ± SD, 300 ± 57 (µg/mL) (*N* = 54), compared to the insufficient vitamin D group, 312 ± 77 (*N* = 379), and the sufficient vitamin D group, 327 ± 83 (*N* = 126), (*p* = 0.003). BP, binding protein.

Using the Global Consensus Recommendations on Prevention and Management of Nutritional Rickets, there was no significant difference in the prevalence of deficient, insufficient, and optimal 25(OH) vitamin D. Further, levels of vitamin D-binding protein were significantly decreased in the deficient vitamin D group compared to the insufficient vitamin D group and the optimal vitamin D group (*p* = 0.007), according to the 2011 Endocrine Clinical Society Practice Guidelines for vitamin D deficiency (Figure 1). Similarly, vitamin D-binding protein levels were significantly decreased in the deficient vitamin D group compared to insufficient and sufficient vitamin D groups (*p* = 0.003), according to the Global Consensus Recommendations on Prevention and Management of Nutritional Rickets vitamin D deficiency (Figure 2).

### 3.3. Genotype Distribution of VDR Polymorphisms

The Hardy–Weinberg Equilibrium (HWE) test was used to determine SNP frequency. Table 4 shows genotype distribution and minor allele frequencies within the study population. The allele percentage was calculated at 26.5% for *Fok1* (*rs2228570*; HWE *p* = 0.1), 28.9% for *Taq1* (*rs731236*; HWE *p* = 0.6, and 39% for *Apa1* (*rs7975232*; HWE *p* = 0.02). In the dominant (GG versus AA + AG), recessive (GG + AG versus AA), and additive models for SNP *rs2228570*, the determinants, sex, age, glycaemic status, MetS, and vitamin D, were not significant (Table 5). However, results from the additive model indicate that obesity was a significant determinant, with a two-fold increase in the GG genotype [OR (95% CI): 2.04 (1.02; 4.10), *p* = 0.045].

Table 6 shows that in the dominant (AA versus GG + AG), recessive (AA + AG versus GG), and additive (AA vs. AG vs. GG) models for the SNP *rs731236*, the determinants, sex, age, glycaemic status, and MetS, were not significant. However, in the recessive model, the SNP *rs731236* was associated with insufficient vitamin D (*p* = 0.04). In the additive model, there was a lower likelihood of patients being overweight [OR (95% CI):0.65 (0.43; 0.97), *p* = 0.04] in the genotype AG, while the genotype AA had a 1.82 likelihood of being vitamin D-insufficient [OR (95% CI):1.82 (1.07; 3.07), *p* = 0.03].

## 4. Discussion

Vitamin D deficiency is one of the most prevalent nutritional deficiencies in the world. Vitamin D, which was previously known to be involved only in calcium homeostasis, is now known to have several other functions in the human body [22]. Subclinical and asymptomatic vitamin D deficiency is associated with increased risk of multiple malignancies, metabolic and cardiovascular diseases, diabetes, and immune disorders [23]. Studies regarding vitamin D supplementation in African populations are limited [24,25]. Over the last decade, low vitamin D levels have emerged as a risk factor for T2D, but this has not been investigated in South African populations. In this community-based study, we examined the association between serum 25-hydroxy vitamin D levels and glycaemic indicators in diabetic, prediabetic, and healthy subjects from a population at high risk of developing diabetes residing in an urban area of Cape Town, South Africa [14]. Due to differences in opinions regarding cut-off levels of vitamin D deficiency [26], we used both the 2011 Endocrine Clinical Society Practice Guidelines and the more recent Global Consensus Recommendations on Prevention and Management of Nutritional Rickets to define vitamin D deficiency as either a 25(OH) vitamin D level below 20 ng/mL (50 nmol/L) or 12 ng/mL (30 nmol/L) [20]. Similar to reports from Germany and Japan, we found a mean 25(OH) vitamin D level of 22 ng/mL, which was within the optimal levels of the Global Consensus Recommendations, but considered to be insufficient according to the Endocrine Society Guidelines [19]. We observed significant differences in the overall prevalence of vitamin D deficiency, with 44.5% of the participants classified as deficient according to the Endocrine Clinical Society Practice Guidelines, but only 5.6% were found to be deficient when using the Global Consensus Recommendations. Only 13% had optimal levels, whilst 44.7% had sub-optimal levels when using the latter criteria.

We observed vitamin D deficiency in subjects with either prediabetes, screen-detected diabetes mellitus, or known diabetes mellitus compared to normoglycaemic subjects using the former criteria. Surprisingly, when using the Global Consensus Criteria, which uses a much lower cut-off to define vitamin D deficiency, the percentage of deficient subjects was similar in each of the glycaemic groups (Table 3). This raises an important question: what levels must we use to define vitamin D deficiency? When comparing our results with earlier studies that have used higher cut-offs endorsed by the Endocrine Society, similar conclusions were arrived at: vitamin D deficiency is associated with prediabetes and diabetes. In a study from Japan, which used a cut-off of 50 nmol/L, vitamin D deficiency was found in 54% of participants, whilst it was 90.9% when a cut-off of 75 nmol/L was used. The high prevalence of vitamin D insufficiency in Japanese populations was attributed to darker skin and rare use of vitamin D supplements [27]. Similarly, studies from Egypt and Bangladesh have reported lower vitamin D levels in T2D patients [28,29] compared to healthy controls. Further research is required regarding the influence of skin colour on vitamin D levels.

A study from China examined if higher plasma 25(OH) vitamin D concentrations were associated with lower risks of diabetes in 82500 participants and further tested the relevance of 25(OH) vitamin D in T2D subjects using genetically instrumented differences in plasma 25(OH) vitamin D concentrations to ascertain causality. The concordant results of both the observational and genetic studies indicate that a higher vitamin D status is associated with a lower risk of diabetes and provide support for a causally protective effect of higher vitamin D in the prevention of T2D [30]. Abbasi et al. showed that subjects with prediabetes and low circulating 25(OH) vitamin D levels were mostly insulin-resistant, had impaired β-cell function, and were most likely to develop T2D [31].

In our study, both obesity and overweight were commonly observed. A higher body mass index (BMI) has been associated with lower vitamin D levels. Obesity affects insulin secretion, tissue sensitivity to insulin, and systemic inflammation, but this may not account for differences seen in the levels of vitamin D deficiency between the glycaemic groups, as BMIs were similar. A meta-analysis that examined 55 observational studies showed an inverse relationship between vitamin D levels and BMI in both diabetic and non-diabetic subjects [32]. Studies in low- and middle-income countries have consistently demonstrated that women have lower average 25(OH) vitamin D levels than their male counterparts, which is largely thought to be due to differences in occupation, clothing, and cultural practices, which predisposes women in these countries to lower vitamin D status and is not related to biological differences in vitamin D metabolism between males and females [33]. Similarly, in this study we found lower vitamin D levels in females than in male study participants, although females displayed higher vitamin D-binding protein levels. These sex differences may partially be attributed to the higher BMIs observed in females. Surprisingly, no sex differences were observed in CRP levels. Several studies have suggested that the lower the vitamin D level, the greater the benefit of supplementation in preventing diabetes [34,35]. Thus, it may be prudent to consider the benefit of vitamin D supplementation in this population group, which is at high risk of developing diabetes.

The *VDR* gene is highly polymorphic, widely distributed, located on chromosome 12q13.1 [36], and controls genes related to bone metabolism, inflammation, oxidative damage, and chronic diseases [37]. Vitamin D and its receptor complex play a role in the regulation of insulin secretion from beta cells [38,39]. *VDR* gene variations are associated with the development, progression, and complications of T2D [40,41]. If the vitamin D-binding protein gene is mutated, vitamin D would decrease in serum and target tissues, although sufficient sun exposure or supplementation may ameliorate this. Four common single-nucleotide polymorphisms of the *VDR* gene have been postulated to be associated with T2D in different ethnic populations, namely *FokI* (*rs2228570*), *BsmI* (*rs1544410*), *ApaI* (*rs7975232*), and *TaqI* (*rs731236*). The full-length human *VDR* gene is ~63.5 kb.

In this study, the *VDR* single-nucleotide polymorphisms *Fok1* (*rs2228570*) and *Taq1* (*rs731236*) were not associated with glycaemic status. *Fok1* was also not associated with vitamin D level, although *Taq1* was associated with insufficient vitamin D. Amongst a population in Saudi Arabia, no significant association between the *Fok1* and *Taq1* single-nucleotide polymorphisms and vitamin D deficiency was observed [42]. A study conducted in Russia showed no difference in serum 25(OH) vitamin D concentration between *Taq1* and *Fok1* genotypes [43]. However, in a population from Bangladesh at high risk of T2D, the *ApaI* polymorphism was associated with insulin secretion and a higher prevalence of vitamin D deficiency. The *ApaI* polymorphism was correlated with fasting blood glucose levels, and glucose intolerance was evident among individuals with symptoms of diabetes at the pre-diagnosis stage [44]. As ethnicity reportedly influences *VDR* gene variations [45], the variations observed in our study may be explained by this and the participants’ exposure to environmental factors [46]. Our results indicate that *Fok1* was associated with obesity, similar to observations in T2D Egyptian patients. Patients with mutant recessive homozygous TT genotype C>T polymorphisms exhibited higher waist circumference and BMIs than individuals with the homozygous CC genotype [46]. In our study, the GG genotype of the *Fok1* polymorphism was associated with a two-fold increased risk of developing obesity, similar to subjects harbouring the T allele in Greece [47].

Our study, like others, is not without limitations. The comparison of vitamin D status between different studies is difficult due to the lack of an evidence-based consensus regarding optimal levels of serum 25(OH) vitamin D, since cut-offs used to evaluate vitamin D status vary across studies. Although serum 25(OH) vitamin D measurement is a valid and commonly used biomarker of vitamin D status, its measurement still lacks standardization. Thus, the measurement of 25(OH) vitamin D differs between studies due to differences in analytical methods, assays, and devices used [48]. There are also seasonal variations in serum 25(OH) vitamin D levels, with the highest levels observed towards the end of summer and lowest levels toward the end of winter, but tracking 25(OH) vitamin D concentration over time reveals that a single measurement of serum 25(OH) vitamin D at a given point provides an estimate of future 25(OH) vitamin D levels [10]. Furthermore, this study did not take dietary choices or physical activity into consideration. As such, behavioural differences may influence the interpretation of the results. Further studies are required to determine the influence of diet in this cohort, as well as in a cohort with similar dietary preferences and levels of physical activity.

## 5. Conclusions

We observed that vitamin D deficiency or insufficiency is relatively common in this mixed-ancestry population from Cape Town. Furthermore, we found that vitamin D levels were decreased in individuals with hyperglycaemia, which was not linked to either vitamin D-binding protein or *Fok1* polymorphisms in the vitamin D receptor gene, although *Taq1* was associated with an insufficient vitamin D status. The mechanisms affecting lower vitamin D levels in individuals with hyperglycaemia require further investigation. Finally, a clinical trial of vitamin D supplementation to either revert prediabetes to normoglycaemia or prevent its progression to diabetes in this population group is highly desirable and recommended.

## Figures and Tables

**Table 1:** Participant characteristics categorized according to sex.

	Total, *N* = 968	Males, *N* = 201	Females, *N* = 767	
	Mean ± SD			*p*-Value
Hyperglycaemia, Yes, N (%)	379 (39.1)	69 (34.3)	310 (40.4)	0.12
DM, Yes, N (%)	204 (21.1)	42 (20.9)	162 (21.1)	Combined 0.14
Pre-DM, Yes, N (%)	175 (18.1)	27 (13.4)	148 (19.3)	
**Vitamin D—using Endocrine Clinical Society Practice Guidelines** **Vitamin D—using Endocrine Clinical Society Practice Guidelines**				
Deficient, N (%)	431 (44.5)	73 (36.3)	358 (46.7)	Combined **0.004**
Insufficient, N (%)	411 (42.5)	90 (44.8)	321 (41.9)	
Optimal, N (%)	126 (13.0)	38 (18.9)	88 (11.5)	
**Vitamin D—using Global Consensus Recommendations on Prevention and Management of Nutritional Rickets**				
Deficient, N (%)	54 (5.6)	3 (1.5)	51 (6.6)	Combined **0.003**
Insufficient, N (%)	379 (39.2)	71 (35.3)	308 (40.2)	
Optimal, N (%)	535 (55.3)	127 (63.2)	408 (53.2)	
**Participant characteristics**				
Age (years)	51 ± 14	52 ± 15	51 ± 14	**<0.0001**
BMI (kg/m^2^)	31 ± 8	26 ± 7	32 ± 8	0.7
WaistC (cm)	97 ± 16	91 ± 17	98 ± 16	**<0.0001**
HipC (cm)	109 ± 16	99 ± 12	112 ± 15	**<0.0001**
WHR	0.9 ± 0.1	0.9 ± 0.1	0.9 ± 0.1	**<0.0001**
Vitamin D (25OH) (ng/mL)	22 ± 7.6	24 ± 8	22 ± 7.5	**0.0006**
Vitamin D BP (µg/mL)	320 ± 80	306 ± 74	323 ± 81	**0.007**
FBG (mmol/L)	5.9 ± 3	5.7 ± 2.5	6 ± 3	0.2
Post 2 HRs BG (mmol/L)	6.9 ± 2.9	6.3 ± 3.5	7 ± 2.7	**0.001**
HbA1c (%)	6.3 ± 1.6	6.1 ± 1.3	6.4 ± 1.7	**0.03**
FBI (mIU/L)	10 ± 10	8 ± 11	11 ± 10	**0.006**
Post 2 HRs BI (mIU/L)	64 ± 57	41 ± 44	70 ± 59	**<0.0001**
Glucose/Insulin ratio	0.9 ± 0.9	1.3 ± 1	0.9 ± 0.9	**<0.0001**
Triglycerides (mmol/L)	1.6 ± 1.5	1.6 ± 1.9	1.5 ± 1.4	0.4
HDL Chol (mmol/L)	1.3 ± 0.3	1.2 ± 0.3	1.3 ± 0.3	**<0.0001**
LDL Chol (mmol/L)	3.3 ± 1	3 ± 1	3.4 ± 1	**<0.0001**
Chol (mmol/L)	5.3 ± 1.2	5 ± 1	5 ± 1.2	**<0.0001**
Chol/HDL ratio	4.3 ± 1.2	4.3 ± 1.3	4.3 ± 1.1	0.6
Gamma GT-S (IU/L)	43 ± 54	48 ± 70	42 ± 49	0.2
Creatinine-S (µmol/L)	65 ± 27	80 ± 37	61 ± 22	**<0.0001**
Creatinine-U (mmol/L)	14 ± 8.3	16 ± 8.7	13 ± 8.1	**<0.0001**
ALT (SGPT) (IU/L)	22 ± 23	25 ± 21	22 ± 24	0.1
AST (SGOT) (IU/L)	25 ± 13	28 ± 15	24 ± 13	**0.002**
MDRD (mL/min/1.73 m^2^)	84 ± 12	84 ± 14	84 ± 12	0.9
Parathormone (pmol/L)	5.5 ± 3.4	4.9 ± 3.7	5.6 ± 3.4	**0.01**
Albumin-S (g/L)	43 ± 3.3	44 ± 3.8	43 ± 3.2	**0.0003**
Calcium corrected (mmol/L)	2.3 ± 0.1	2.3 ± 0.1	2.3 ± 0.1	**0.0003**
Calcium-S (mmol/L)	2.4 ± 0.1	2.4 ± 0.1	2.4 ± 0.1	0.5
Phosphate-S (mmol/L)	1.1 ± 0.2	1 ± 0.2	1.1 ± 0.2	**<0.0001**
Sodium-U (mmol/L)	109 ± 54	121 ± 57	105 ± 53	**0.0002**
CRP (mg/L)	8.8 ± 16	8.8 ± 18	8.7 ± 16	0.96
Cotinine (ng/mL)	128 ± 161	159 ± 159	120 ± 160	**0.002**
Smoking, Yes, N (%)	408 (42.2)	111 (55.2)	297 (38.8)	**<0.0001**

DM, diabetes mellitus; BMI, body mass index; WaistC, waist circumference; HipC, hip circumference; WHR, waist-to-hip-ratio; Vitamin D BP, vitamin D binding protein; FBG, fasting blood glucose; BG, blood glucose; HbA1c, glycated haemoglobin; FBI, fasting blood insulin; BI, blood insulin; HDL, high-density lipoprotein; LDL, low-density lipoprotein; Chol, cholesterol; GT-S glutamyl transferase-serum; ALT, alanine aminotransferase; SGPT, serum glutamic-pyruvic transaminase; AST, aspartate aminotransferase; SGOT, serum glutamic-oxaloacetic transaminase; MDRD, modification of diet in renal disease; CRP, c-reactive protein. Bold *p* values indicates significant differences in the analysed data.

**Table 2:** Vitamin D deficiency, insufficiency, and optimal level cut-offs used to assess vitamin D status.

Vitamin D (ng/mL), Total Group *N* = 968					
Vitamin D—Endocrine Clinical Society Practice Guidelines for vitamin D deficiency			Vitamin D—Global Consensus Recommendations on Prevention and Management of Nutritional Rickets		
**Category**	**Cut-off value (ng/mL)**	***N*** **(%)**	**Category**	**Cut-off value (ng/mL)**	***N*** **(%)**
Deficiency	<20	431 (44.5%)	Deficiency	<12	54/968 (5.6%)
Insufficiency	20–29	411 (42.5%)	Insufficiency	12 to 20	379/968 (39.2%)
Optimal levels	≥30	126 (13.0%)	Optimal levels	>20	535/968 (55.3%)

**Table 3:** Participant characteristics according to glycaemic status.

	Total Group, *N* = 968						
	Normo-Glycaemic, *N* = 589	Pre-Diabetes Mellitus *N* = 175	Screen-Detected Diabetes Mellitus, *N* = 64	Known Diabetes Mellitus, *N* = 140	Gender	Diagnosis	Gender * Diagnosis
		Mean ± SD			*p*-Value	*p*-Value	*p*-Value
**Vitamin D—using Endocrine Clinical Society Practice Guidelines for vitamin D deficiency**							
Deficient, N (%)	240 (40.7)	89 (50.9)	31 (48.4)	71 (50.7)			Combined 0.009 *
Insufficient, N (%)	255 (43.3)	68 (38.9)	30 (46.9)	58 (41.4)			
Optimal, N (%)	94 (16.0)	18 (10.3)	3 (4.7)	11 (7.9)			
**Vitamin D—using Global Consensus Recommendations on Prevention and Management of Nutritional Rickets**							
Deficient, N (%)	31 (5.3)	11 (6.3)	5 (7.8)	7 (5.0)			Combined 0.17 *
Insufficient, N (%)	211 (35.8)	78 (44.6)	26 (40.6)	64 (45.7)			
Optimal, N (%)	347 (58.9)	86 (49.1)	33 (51.6)	69 (49.3)			
**Participant characteristics**							
Age (years)	48 ± 14	56 ± 14	57 ± 11	58 ± 11	0.2	**<0.0001**	0.8
BMI (kg/m2)	29 ± 8	32 ± 8	34 ± 7.5	32 ± 7	**<0.0001**	**<0.0001**	0.3
WaistC (cm)	93 ± 16	101 ± 15	105 ± 14	103 ± 14	**0.006**	**<0.0001**	0.07
HipC (cm)	107 ± 15	112 ± 16	113 ± 15	111 ± 15	**<0.0001**	**0.007**	0.6
WHR	0.9 ± 0.1	0.9 ± 0.1	0.9 ± 0.1	0.9 ± 0.1	**<0.0001**	**<0.0001**	**0.008**
Vitamin D (25OH) (ng/mL)	23 ± 8	21 ± 7.3	20 ± 6.2	21 ± 6.2	0.2	**0.002**	**0.02**
Vitamin D BP (µg/mL)	321 ± 81	322 ± 74	312 ± 81	317 ± 81	**0.003**	0.4	0.3
FBG (mmol/L)	4.8 ± 0.5	5.3 ± 0.6	7.7 ± 4	10.6 ± 4.8	0.8	**<0.0001**	**<0.0001**
Post 2 HRs BG (mmol/L)	5.6 ± 1.3	8.9 ± 1.1	14 ± 3.7	NA	**0.001**	**<0.0001**	**<0.0001**
HbA1c (%)	5.6 ± 0.4	6 ± 0.5	7.2 ± 1.8	9.2 ± 2.3	**0.002**	**<0.0001**	**<0.0001**
FBI (mIU/L)	8.2 ± 6.5	12 ± 13	14 ± 14	14 ± 16	0.7	**<0.0001**	**0.03**
Post 2 HRs BI (mIU/L)	52 ± 49	100 ± 70	70 ± 46	NA	**0.002**	**<0.0001**	0.9
Glucose/Insulin ratio	0.9 ± 0.6	0.8 ± 0.6	0.9 ± 1.1	1.5 ± 1.6	**0.01**	**<0.0001**	**0.0006**
Triglycerides (mmol/L)	1.3 ± 1	1.8 ± 2.5	2.1 ± 2.3	2.1 ± 1.4	**0.001**	**<0.0001**	**<0.0001**
HDL Chol (mmol/L)	1.3 ± 0.3	1.3 ± 0.3	1.3 ± 0.3	1.2 ± 0.3	**0.0005**	0.09	0.9
LDL Chol (mmol/L)	3.2 ± 1	3.5 ± 1	3.6 ± 1	3.4 ± 1.1	**0.02**	**0.008**	0.1
Chol (mmol/L)	5.2 ± 1.2	5.5 ± 1.1	5.6 ± 1.2	5.4 ± 1.3	**0.02**	**0.002**	**0.02**
Chol/HDL ratio	4.2 ± 1.2	4.4 ± 1.1	4.6 ± 1.2	4.6 ± 1.2	0.2	**0.0003**	0.07
Gamma GT-S (IU/L)	41 ± 63	42 ± 33	53 ± 40	47 ± 41	0.7	0.8	0.6
Creatinine-S (umol/L)	63 ± 21	64 ± 22	67 ± 36	71 ± 45	**<0.0001**	**0.001**	0.1
Creatinine-U (mmol/L)	14 ± 8	14 ± 9.2	16 ± 9.8	12 ± 7.5	**0.002**	0.2	0.1
ALT (SGPT) (IU/L)	21 ± 21	22 ± 12	34 ± 58	22 ± 15	0.9	0.3	0.2
AST (SGOT) (IU/L)	25 ± 13	24 ± 7.6	31 ± 27	23 ± 11	0.9	0.3	**0.02**
MDRD (mL/min/1.73 m^2^)	85 ± 11	83 ± 12	83 ± 15	80 ± 17	0.3	**0.006**	0.7
Parathormone (pmol/L)	5.3 ± 3.3	5.5 ± 3	6.2 ± 4	5.8 ± 4	0.9	**0.02**	0.09
Albumin-S (g/L)	43 ± 3.5	43 ± 2.8	42 ± 2.8	43 ± 3.3	**0.02**	0.5	0.6
Calcium corrected (mmol/L)	2.3 ± 0.1	2.3 ± 0.1	2.3 ± 0.1	2.3 ± 0.1	**0.002**	0.2	0.05
Calcium-S (mmol/L)	2.4 ± 0.1	2.4 ± 0.1	2.4 ± 0.1	2.4 ± 0.1	0.3	0.7	0.4
Phosphate-S (mmol/L)	1.1 ± 0.2	1.1 ± 0.2	1.1 ± 0.2	1.1 ± 0.2	**0.002**	0.7	0.4
Sodium-U (mmol/L)	116 ± 56	104 ± 54	90 ± 46	93 ± 44	0.08	**<0.0001**	0.1
CRP (mg/L)	7.4 ± 15	10 ± 15	14 ± 21	11 ± 22	0.2	**0.002**	0.2
Cotinine (ng/mL)	142 ± 163	130 ± 164	77 ± 134	92 ± 150	0.3	**0.0001**	0.2
Smoking, Yes % (N)	280 (47.7)	74 (42.3)	16 (25)	38 (27.1)	-	-	<0.0001 *

* denotes data analysis according to gender; Bold *p* values indicates significant differences in the analysed data.

**Table 4:** Genotype distributions and minor allele frequencies (HWE: Hardy–Weinberg Equilibrium).

Fok1 Rs2228570		Taq1 rs731236		Apa1 rs7975232	
G/G, *n* (%)	532/968 (55)	A/A, *n* (%)	486/968 (50.2)	A/A, *n* (%)	377/968 (38.9)
A/G, *n* (%)	358 (37)	A/G, *n* (%)	404 (41.7)	A/C, *n* (%)	426 (44)
A/A, *n* (%)	78 (8.1)	G/G, *n* (%)	78 (8.1)	C/C, *n* (%)	165 (17)
A, *n* (%)	514 (26.5)	G, *n* (%)	560 (28.9)	C, *n* (%)	756 (39)
HWE (*p*-value)	0.1	HWE (*p*-value)	0.6	HWE (*p*-value)	**0.02**

Bold *p* values indicates significant differences in the analysed data.

**Table 5:** Logistic regression for dominant, recessive, and additive models for single-nucleotide polymorphisms *rs2228570*.

	Dominant		Recessive		Additive					
					AG		GG		AA	
	OR (95% CI)	*p*-Value	OR (95% CI)	*p*-Value	OR (95% CI)	*p*-Value	OR (95% CI)	*p*-Value	OR (95% CI)	*p*-Value
**Sex**										
Male	1		1		1		1		1	
Female	1.12 (0.79; 1.6)	0.5	1.32 (0.7; 2.5)	0.4	1.26 (0.64; 2.48)	0.5	1.36 (0.7; 2.62)	0.4	1.08 (0.74; 1.56)	0.7
**Age**	1 (0.99; 1.01)	0.4	1.01 (0.99; 1.03)	0.2	0.99 (0.97; 1.01)	0.3	0.99 (0.97; 1.01)	0.2	1 (0.99; 1.01)	0.6
**Glycaemic status**										
Normal	1		1		1		1		1	
Pre-DM	0.85 (0.59; 1.22)	0.4	0.81 (0.4; 1.65)	0.6	1.14 (0.54; 2.41)	0.7	1.31 (0.63; 2.72)	0.5	0.87 (0.59; 1.27)	0.5
DM	0.8 (0.56; 1.15)	0.2	1.13 (0.6; 2.14)	0.7	0.74 (0.38; 1.46)	0.4	0.98 (0.51; 1.88)	1	0.75 (0.51; 1.11)	0.2
**JIS Criteria**										
No	1		1		1		1		1	
Yes	0.81 (0.6; 1.11)	0.2	0.97 (0.55; 1.72)	0.9	0.9 (0.49; 1.64)	0.7	1.13 (0.63; 2.02)	0.7	1.25 (0.91; 1.73)	0.2
**Obesity status**										
Normal	1		1		1		1		1	
Overweight	0.92 (0.63; 1.36)	0.7	0.82 (0.43; 1.59)	0.6	1.18 (0.59; 2.37)	0.6	1.24 (0.63; 2.45)	0.5	0.95 (0.63; 1.44)	0.8
Obese	0.81 (0.55; 1.19)	0.3	0.51 (0.26; 1)	0.1	1.85 (0.9; 3.78)	0.1	2.04 (1.02; 4.1)	0.045	0.9 (0.6; 1.35)	0.6
**Vitamin D/ES**										
Sufficient	1		1		1		1		1	
Insufficient	1.16 (0.88; 1.52)	0.3	1.26 (0.77; 2.07)	0.4	0.85 (0.5; 1.44)	0.5	0.76 (0.45; 1.26)	0.3	1.12 (0.84; 1.5)	0.4
Deficient	0.81 (0.44; 1.48)	0.5	1.15 (0.39; 3.42)	0.8	0.73 (0.23; 2.34)	0.6	0.96 (0.32; 2.9)	0.9	0.76 (0.4; 1.46)	0.4

**Table 6:** Logistic regression for dominant, recessive, and additive models for single-nucleotide polymorphisms *rs731236*.

	Dominant		Recessive		Additive					
					AG		GG		AA	
	OR (95% CI)	*p*-Value	OR (95% CI)	*p*-Value	OR (95% CI)	*p*-Value	OR (95% CI)	*p*-Value	OR (95% CI)	*p*-Value
**Sex**										
Male	1		1		1		1		1	
Female	0.83 (0.59; 1.18)	0.3	1.32 (0.66; 2.66)	0.4	1.28 (0.89; 1.84)	0.2	0.84 (0.41; 1.73)	0.6	0.66 (0.32; 1.36)	0.3
**Age**	1.01 (1; 1.02)	0.1	1 (0.98; 1.02)	0.9	1.01 (1; 1.02)	0.1	1 (0.98; 1.02)	0.8	0.99 (0.98; 1.01)	0.6
**Glycaemic status**										
Normal	1		1		1		1		1	
Pre-DM	1.12 (0.78; 1.61)	0.5	0.54 (0.25; 1.15)	0.1	1.25 (0.86; 1.82)	0.2	0.6 (0.27; 1.3)	0.2	0.48 (0.22; 1.05)	0.06
DM	1.09 (0.76; 1.55)	0.6	0.85 (0.44; 1.62)	0.6	1.13 (0.78; 1.64)	0.5	0.9 (0.46; 1.75)	0.7	0.79 (0.4; 1.56)	0.5
**JIS Criteria**										
No	1		1		1		1		1	
Yes	1.17 (0.86; 1.58)	0.3	0.99 (0.56; 1.74)	1	1.19 (0.86; 1.65)	0.3	1.06 (0.59; 1.9)	0.8	0.89 (0.49; 1.62)	0.7
**Obesity status**										
Normal	1		1		1		1		1	
Overweight	0.68 (0.46; 1.01)	0.06	1.09 (0.52; 2.28)	0.8	0.65 (0.43; 0.97)	0.04	0.91 (0.42; 1.95)	0.8	1.41 (0.64; 3.08)	0.4
Obese	1 (0.68; 1.46)	1	1.19 (0.58; 2.44)	0.6	0.97 (0.65; 1.44)	0.9	1.18 (0.56; 2.46)	0.7	1.22 (0.58; 2.58)	0.6
**Vitamin D**										
Sufficient	1		1		1		1		1	
Insufficient	0.96 (0.73; 1.27)	0.8	1.68 (1.02; 2.77)	0.04	0.87 (0.65; 1.16)	0.3	1.58 (0.94; 2.65)	0.09	1.82 (1.07; 3.07)	0.03
Deficient	0.87 (0.48; 1.56)	0.6	1.61 (0.59; 4.37)	0.4	0.78 (0.41; 1.46)	0.4	1.44 (0.51; 4.05)	0.5	1.86 (0.64; 5.41)	0.3

## Data Availability

The sequence data used to support the findings of this study have been deposited in the SRA BioProject database (accession number: PRJNA723337).
